# Bacteria from nodules of *Abrus mollis* Hance: genetic diversity and screening of highly efficient growth-promoting strains

**DOI:** 10.3389/fmicb.2024.1345000

**Published:** 2024-04-12

**Authors:** Kexin Cao, Jianhua Chen, Qiuling Li, Peng Gu, Liangbo Li, Rongshao Huang

**Affiliations:** ^1^College of Agriculture, Guangxi University, Nanning, Guangxi, China; ^2^College of Pharmacy, Guangxi University of Chinese Medicine, Nanning, Guangxi, China

**Keywords:** *Abrus mollis* Hance., root nodule, endophytic bacteria, microbial diversity, rhizobium

## Abstract

**Introduction:**

*Abrus mollis* Hance. (AM) is an important species used in southern Chinese medicine. It is mainly found in Guangdong and Guangxi provinces in China, and it is effective in the treatment of hepatitis. Endophytic bacteria are known to affect the growth and quality of medicinal plants. However, there are limited reports describing endophytic bacteria related to AM.

**Methods:**

In the present study, Illumina-based 16S rRNA gene sequencing was used to investigate the endophytic bacterial communities of root nodules of AM at five sampling sites in Guangxi. In addition, 179 strains of endophytic bacteria were isolated and categorized into 13 haplotypes based on recA sequence analysis.

**Results:**

The phylogeny of the 16S rRNA gene sequences revealed a predominance of nonrhizobial endophytes. Microbial diversity analysis showed that Proteobacteria was the dominant phylum in all samples, while Bradyrhizobium was the dominant genus in different samples. An efficient strain, *Rhizobium tropici* FM-19, was screened and obtained through greenhouse experiments. The AM plants inoculated with this strain showed the best growth performance and high nitrogen fixation and nodulation capacity. Notably, total phenols and total flavonoids, important active components in AM, increased by 30.9 and 42.7%, respectively, after inoculation with *Rhizobium tropici* FM-19.

**Discussion:**

This study provides insights into the complex microbial diversity of AM nodules and provides strain information for the efficient cultivation of AM.

## 1 Introduction

The genus *Abrus* is a member of the legume family, with 12 species distributed throughout the world and four species in China, namely, *Abrus cantoniensis* Hance., *Abrus mollis* Hance., *Abrus pulchellus* Wall. and *Abrus precatorius* Linn. *Abrus mollis* Hance. (AM) is used in Chinese herbal medicine and is mainly distributed in Guangdong and Guangxi (Cao et al., 2023). It is also the main raw material for the hepatitis treatment drug “Jigucao capsule.” People often use AM as an ingredient in soups and herbal teas to promote health (Fu et al., 2018). AM has been known to cure liver diseases, such as liver injury (Yuan et al., 2014), hepatitis (Hu et al., 2019), nonalcoholic fatty liver (Yan et al., 2016) and other diseases, in clinical applications.

Nitrogen is one of the most important nutrients needed for plant growth. Adequate nitrogen availability can help plants flourish and improve photosynthesis, which plays a vital role in plant growth and development (Ahluwalia et al., 2021). Some nitrogen-fixing microorganisms in nature can convert nitrogen into forms that can be absorbed and utilized by plants, a process known as biological nitrogen fixation (BNF) (Singh et al., 2022). Rhizobial symbiotic nitrogen fixation, as the most efficient nitrogen fixation system in nature, fixes approximately 40 million tons of pure nitrogen annually, accounting for 65% of total biological nitrogen fixation (Herridge et al., 2018). As the most valuable nitrogen fixation system, rhizobial symbiotic nitrogen fixation has been widely applied in many countries where soybeans and forages are grown, and a significant reduction in the use of chemical nitrogen fertilizers has been achieved (Guan et al., 2012). The number of newly discovered rhizobia is increasing, but the studies are concentrated in common crops such as soybean and alfalfa. In contrast, relatively little research has been done on leguminous medicinal plants. With the increasing emphasis on the development of traditional Chinese medicine (TCM) in China, research on rhizobia in leguminous medicinal plants has been increasing. In recent years, an increasing number of scholars have studied the rhizobial symbiotic system of leguminous medicinal plants. For these plants, the nitrogen produced by BNF in the plant participates in primary and secondary metabolic pathways to promote the synthesis of active ingredients (Feng et al., 2021). Tang et al. (2017) inoculated the legume *Sophora tonkinensis* Gagnep. with rhizobia, and the plants showed significant increases in the major components matrine and oxmatrine. In addition, the nitrogen fixation effect of the rhizobial symbiosis system can satisfy the nitrogen demand for medicinal plant cultivation, and at the same time, it can effectively improve plant resistance to drought, low temperature and salt stress (Jiang et al., 2019). Therefore, it is very important to study the leguminous medicinal plant–rhizobial nitrogen fixation system to discover new rhizobial resources, to screen efficient related strains and to apply the strains in agricultural production. By utilizing the full capacity of rhizobial resources in medicinal plants, the green, healthy and sustainable development of medicinal plants can be ensured in the future.

The study of endophytic of root nodules is mainly based on the traditional microbial culture method. This method is mainly used by homogenizing the root nodules and inoculating onto isolation media in order to obtain pure cultures of endophytic. Finally, morphological and molecular characterization were performed to determine the taxonomic status of the strains. This method is very simple and convenient, however, some microorganisms are difficult or unculturable to culture. So it may lead to a large amount of missing information about root nodules endophytic, and the development of high-throughput sequencing technology provides a new method to solve this research bottleneck. Currently, high-throughput sequencing technology is less used in root nodules endophytes diversity. Zhang et al. (2021) investigated the diversity of endophytes in *Hippophae tibetana* root nodules using high-throughput sequencing technology, and found that *Frankia* were the dominant taxa in *Hippophae tibetana* root nodules. However, high-throughput sequencing technology has not been applied to the study of endophytes diversity in AM root nodules.

In this study, high-throughput sequencing was used to evaluate the endophytic bacterial community and diversity of AM nodules in different regions of Guangxi Province, China. In addition, the soil physicochemical properties of different AM habitats were determined and analyzed to reveal the effects of different ecological environments on the diversity of endophytic bacteria in AM rhizobia. Finally, a greenhouse experiment was conducted to determine growth and physiological and biochemical changes in the inoculated plants, to explore the growth-promoting effect of rhizobia on AM and to screen for efficient growth-promoting strains. This work may enable the exploration of rhizobial resources specific to AM and contribute to the efficient development of rhizobium–AM symbiosis.

## 2 Materials and methods

### 2.1 Root nodule collection

Root nodules were sampled in June 2022 from AM plants in Lingshan County (LS), Pingnan County (PN), Fumian County (FM), Yuzhou County (YZ) and Nanning City (Nn) in Guangxi Province. The details of the five root nodule collection sites and soil physicochemical properties are shown in Table 1. Five points were selected as representative of each sampling site and the soil was carefully dug through the AM roots using a shovel. About 20 g of large and full, fresh root nodules were collected from each site. At the same time, about 200 g of soil within 5 cm of the roots and 10 cm deep was collected and put into a sealed bag for physicochemical characterization. The root nodules were transported in an ice box to a laboratory and stored at 4°C until further use.

**Table 1:** Details and soil physicochemical properties of the root nodule sites.

	LS	PN	FM	YZ	Nn
Altitude (m)	70	70	118	78.5	100
longitude	109.1349	110.2646	109.5821	110.0331	108.3801
latitude	22.2329	23.4638	22.3246	22.8621	22.394
pH	4.1	4.8	5.0	4.7	5.0
Organic matter (g/kg)	53.3	37.5	35.0	33.0	19.6
Total P (%)	0.069	0.102	0.127	0.035	0.053
Total K (%)	0.93	1.39	1.11	2.19	0.15
Hydrolysable N (mg/kg)	100.1	97.3	93.9	83.5	81.7
Available P (mg/kg)	43.6	220.5	88.5	2.2	174.5
Available K (mg/kg)	126	135	236	170	102

### 2.2 DNA extraction

The root nodules were surface-sterilized using methods reported by Du et al. (2021). Specifically, the root nodules were washed thoroughly with sterile water, immersed in 70% ethanol for 3 min, washed with fresh sodium hypochlorite solution (2.5% available Cl^−^) for 5 min with agitation, rinsed three times with 70% ethanol for 30 s, and finally washed five times with sterile distilled water. Fifty microliters of the final rinse water was applied to LB medium, and the medium was examined for bacterial growth after incubation at 30°C for 72 h. If there was no bacterial growth, the surface disinfection procedure was confirmed to be effective, and the samples were used for further analysis.

Total genomic DNA from samples was extracted using the CTAB method. DNA concentration and purity were monitored on 1% agarose gels. According to the concentration, DNA was diluted to 1 ng/μL using sterile water.

### 2.3 Amplicon library preparation and sequencing

Purified amplicons were pooled in equimolar ratios and paired-end sequenced by Novogene Co., Ltd. (Beijing, China) using an TruSeq® DNA PCR-Free Sample Preparation Kit (Illumina, United States) following manufacturer’s recommendations. Raw 16S rRNA gene sequencing reads were demultiplexed, quality-filtered by fastq version 0.20.0, and merged by FLASH version 1.2.7. Operational taxonomic units (OTUs) with a 97% similarity cut-off were clustered using Uparse v7.1, and chimeric sequences were identified and removed. The taxonomy of each OTU representative sequence was analyzed by RDP Classifier version 2.2 against the 16S rRNA database using a confidence threshold of 0.7.

### 2.4 Data analysis

Alpha diversity was calculated including Chao, ACE, Shannon, and Simpson indices using QIIME software (Version 1.7.0). The sequences were clustered into operational taxonomic units (OTUs) at a similarity level of 97% by UPARSE to generate rarefaction curves and to calculate the richness and diversity indices (Gu et al., 2018). The Ribosomal Database Project (RDP) Classifier tool was used to classify all sequences into different taxonomic groups (Wang et al., 2007). β-diversity was visualized using principal coordinates analysis (PCoA) and non-metric multidimensional scaling (NMDS) based on the distance matrix, with the calculation of the Euclidean and BrayCurtis algorithm, respectively. To investigate similarities among different samples, OTU information was analyzed using Bray–Curtis-based principal coordinate analysis (PCoA) (Zhang et al., 2019). Representative sequences of OTUs were compared and analyzed by the RDP Classifier algorithm at each level (phylum, class, family, and genus) (Jiang et al., 2013). The linear discriminant analysis effect size (LEfSe) technique was used to identify biomarkers with statistically significant differences among groups (Segata et al., 2011). RDA was used to analyze the relationships between the bacterial community and environmental factors (Wang et al., 2015).

### 2.5 Rhizobium isolation and culture conditions

For rhizobium isolation, root nodules collected from each sampling point were rinsed with running water until adhering soil particles were completely removed, and the root nodules were then washed with distilled water. The root nodules were immersed in 95% ethanol for 30 s to eliminate surface tension, the ethanol was poured off, and the samples were immersed in 0.2% HgCl_2_ solution for 5 min and finally rinsed 5–6 times with sterile water. Root nodules (0.5 g) were crushed with 4.5 mL of sterile water under aseptic conditions. The suspensions were diluted by a gradient to 10^−3^, and 100 μL of suspension was plated on yeast extract mannitol agar (YMA: mannitol 10.0 g, yeast extract 3.0 g, K_2_HPO_4_ 0.25 g, KH_2_PO_4_ 0.25 g, MgSO_4·_7H_2_0 0.2 g, NaCl 0.1 g, agar 10 g, and distilled water 1,000 mL; pH 7.2). All the inoculated YMA plates were cultured upside down at 28°C until single colonies appeared. Then, the purified cultures were suspended in sterilized 50% (*v/v*) glycerol and maintained at-80°C for long-term storage.

### 2.6 Symbiotic characteristics of the isolates

The genomic DNA of each isolate was extracted using a TIANGEN genomic DNA extraction kit for bacteria (TIANGEN, China) and was used as the template to amplify *rec A* sequences with the primer pair rec A 41F/640R (Liu et al., 2021). The amplification products were sequenced by Beijing Tsingke Biotechnology Co., Ltd. All the obtained sequences were aligned using ClustalW integrated in MEGAX, and the *recA* haplotypes were classified through cluster analysis to select representative strains (Li et al., 2015). Each representative strain of AM plants was tested in greenhouse-based pot experiments. The AM seeds were soaked in 0.1% Hg_2_Cl solution for 10 min for surface disinfection, rinsed 5–6 times with sterile water and then cooled naturally in 50°C distilled water. Once the seeds had imbibed the water, they were placed in a sterile germination box and germinated in a 25°C incubator. The seedlings were repotted into pots with sterilized substrate (vermiculite:seedling substrate = 1:2) once they reached a height of 1–2 cm and were inoculated with the desired rhizobial liquid inoculum (suspended in distilled water, 10^8^ cells/mL) after true leaves had formed. Each plant was inoculated with 10 mL of rhizobial liquid inoculum every 10 days after the first inoculation for a total of 3–5 times. Plants inoculated with distilled water were included as controls. Each treatment was repeated three times. Sixty days after inoculation, the plants were removed from the soil and rinsed with tap water.

### 2.7 Agronomic traits and physicochemical analysis of plants

Agronomic parameters such as fresh weight, dry weight, and nodule weight after AM plant harvest were observed. The roots of fresh plants were cut from the first true leaves, and the fresh weights of the aboveground and underground parts of the plants were measured. The plants were then dried in an oven until they reached a constant weight, and the dry weight of the plants was recorded. For nodulation data, the mature nodules of each plant were washed with distilled water and weighed, and an average was computed. Analysis of variance (ANOVA) was performed on the growth and biochemical parameter data. Duncan’s test (IBM SPSS Statistics 19, United States) was used to test for significant differences between treatment means.

## 3 Results

### 3.1 OTU composition of the bacterial community

After read-quality filtering, a total of 1,501,042 high-quality sequences (clean tags) remained and were queried. The total number of bases was 538,864,653, and the average read length was 409 bp. In addition, to obtain the taxonomic information corresponding to each OTU, the RDP Classifier algorithm was used to annotate OTUs at the phylum to genus level (Table 2).

**Table 2:** The endophytic bacterial OTU composition of the five root nodule locations.

Sample	Phylum	Class	Order	Family	Genus
PN	35	77	174	231	334
Nn	35	78	162	229	332
LS	36	81	172	258	353
YZ	21	42	93	136	199
FM	40	89	178	246	332

Rarefaction curves, combined with the estimated coverage values, suggested that the data were sufficient to capture most of the bacterial diversity in the samples. To visualize the differences in OTUs in root nodules from different origins, a detailed Venn diagram of the distribution of common or unique OTUs from different origins was constructed, which is shown in Supplementary Figure S1. The number of OTUs obtained was highest in PN, followed by FM, while the lowest number of OTUs was present in YZ. OTUs common to root nodules taken from the five locations represented 6.69% (315) of the total number of OTUs, and the remaining OTUs were shared between or unique to the samples. PN contained the most specific OTUs, accounting for 9.84% (463) of its total OTUs, indicating that PN had the highest abundance of specific bacterial groups. Furthermore, PN and FM jointly had 2044 OTUs, 71.15 and 71.95% of their total OTUs, respectively, indicating that more bacterial taxa were shared between them than were unique. Nn and FM shared 1,200 OTUs, accounting for 62.73 and 42.24%, respectively, showing similarities between the two. LS and YZ jointly had 560 OTUs, accounting for 22.24 and 59.51% of their total OTUs, respectively, suggesting similarities in these two communities.

### 3.2 Alpha diversity analysis of bacterial communities

To further assess the abundance and diversity of the endophytic bacterial community in root nodules, the alpha diversity of samples was calculated, and the results are detailed in Table 3. The alpha diversity indices included the Shannon, Simpson and Chao1 indices. The Chao1 index was used to estimate the abundance of species, with a larger value indicating higher community richness (Baláž and Balážová, 2012). The Chao1 index indicated that the abundance of bacterial communities was in the order PN > FM > LS > Nn > YZ. The Shannon index was used to evaluate bacterial diversity, where the larger the values were, the higher the community diversity (Braga and Diniz, 2015). The Shannon index of the bacterial community in PN was the highest, followed by that in LS, while the Shannon index in FM was the lowest. The Simpson index was used to characterize the diversity and uniformity of the species distribution within a community, with greater values indicating higher community diversity (Chen et al., 2022). The Simpson index indicated that the bacterial community diversity was in the order PN > LS > YZ > Nn > FM. This result was consistent with that of the Shannon index, but it was slightly different from that of the Chao1 index.

**Table 3:** Operational taxonomic unit (OTU; 97% similarity cutoff) richness and diversity indices of different samples associated with root nodules.

Sample	OTUs observed	Shannon index	Simpson index	Chao 1 index	Coverage (%)
PN	1901.67 ± 133.52^a^	5.94 ± 0.62^a^	0.85 ± 0.04^a^	2364.20 ± 94.55^a^	98.90%
Nn	844.67 ± 153.56^bc^	2.58 ± 0.23^b^	0.47 ± 0.04^bc^	1095.11 ± 147.47^bc^	99.40%
LS	1294.00 ± 340.20^ab^	4.57 ± 0.13^a^	0.62 ± 0.04^b^	1692.33 ± 425.56^ab^	99.10%
YZ	521.33 ± 53.07^c^	2.63 ± 0.40 ^b^	0.56 ± 0.06^bc^	656.10 ± 70.47 ^c^	99.70%
FM	1478.33 ± 337.34^ab^	2.32 ± 0.67^b^	0.40 ± 0.08^c^	1985.34 ± 399.55^ab^	98.93%

Differences in bacterial alpha diversity based on a 16S rDNA sequence assignment dataset with a 97% sequence similarity threshold in upland root nodules. Values were tested using analysis of variance (ANOVA) with three replicates in each treatment. Data for the same diversity index followed by the same lowercase letter are not significantly different at the *p* = 0.05 level.

### 3.3 Beta diversity analysis of bacterial communities

**Figure 1:**
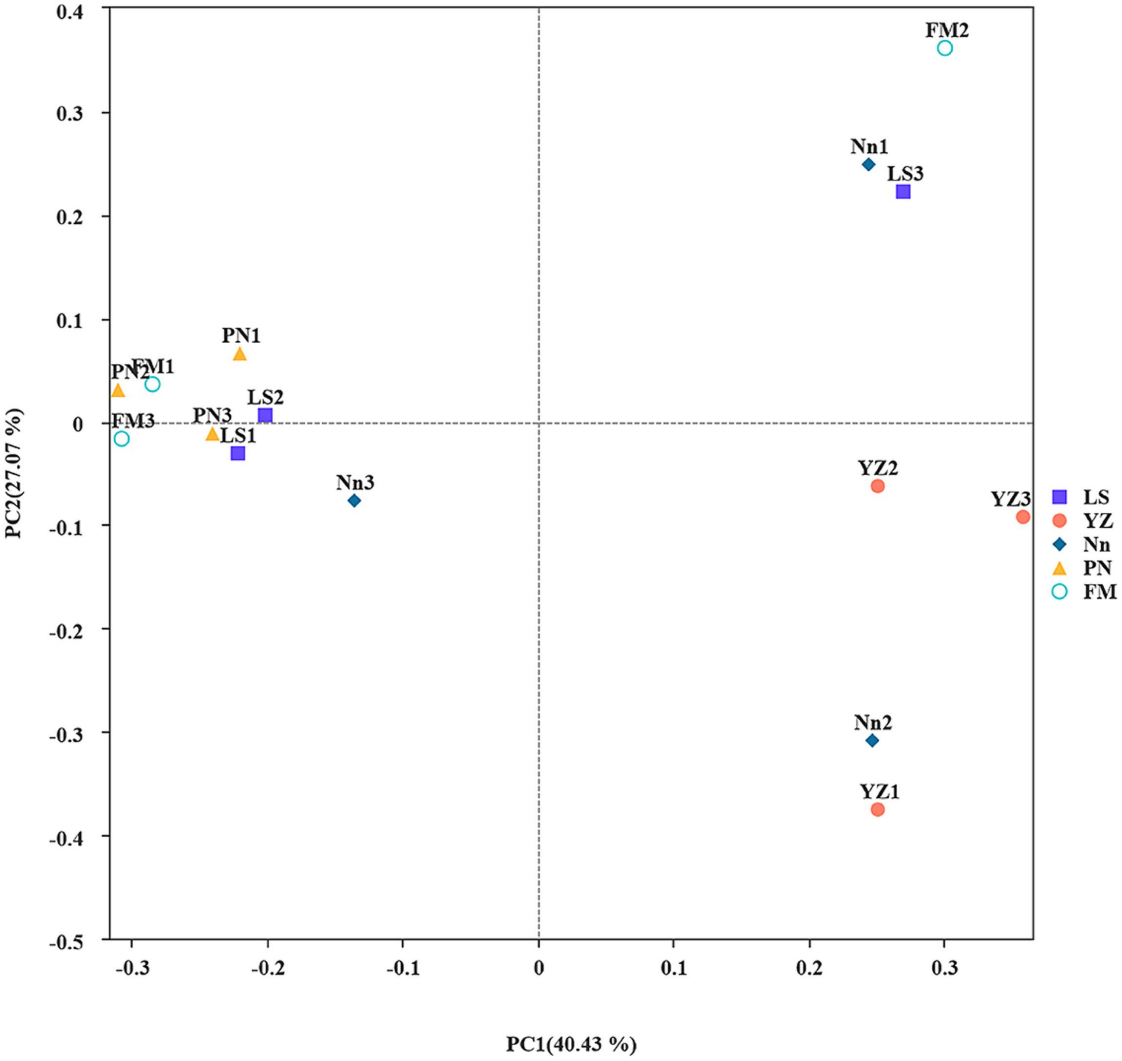
Principal coordinate analysis (PCoA): Ecological differences between the different groups and samples in the case of mixed samples at the level of 97% sequence similarity. The abscissa and ordinates represent the contribution rates of principal components 1 and 2, respectively, to the distribution of the samples. Each point in the figure represents a sample, and points of the same color come from the same group.

The similarity or difference in the root nodule community structure of plants from different origins was studied by using the Bray–Curtis-based PCoA method (Yue et al., 2022). To investigate differences in endophytic bacterial community structure in root nodules taken from the 5 locations, PCoA was used to map the two-dimensional distribution of root nodules, as shown in Figure 1. The distance between each pair of samples reflects the similarity of their community structures. PCoA 1 and PCoA 2 explained 40.43 and 27.07% of the variation, respectively. The PCoA results showed that the distances between samples LS, FM and PN were short, which indicated that the structures of the endophytic bacterial communities in samples LS, FM and PN were similar. Sample YZ was obviously separated from the other samples, indicating that its community structure was very different, and the same was true for its alpha diversity. This may have occurred due to the unique growth environment of AM. The soil organic matter content of YZ was lowest among the five sample collection sites, which resulted in a decrease in the type and quantity of bacterial communities.

### 3.4 Microbial taxonomic analysis at the phylum level

**Figure 2:**
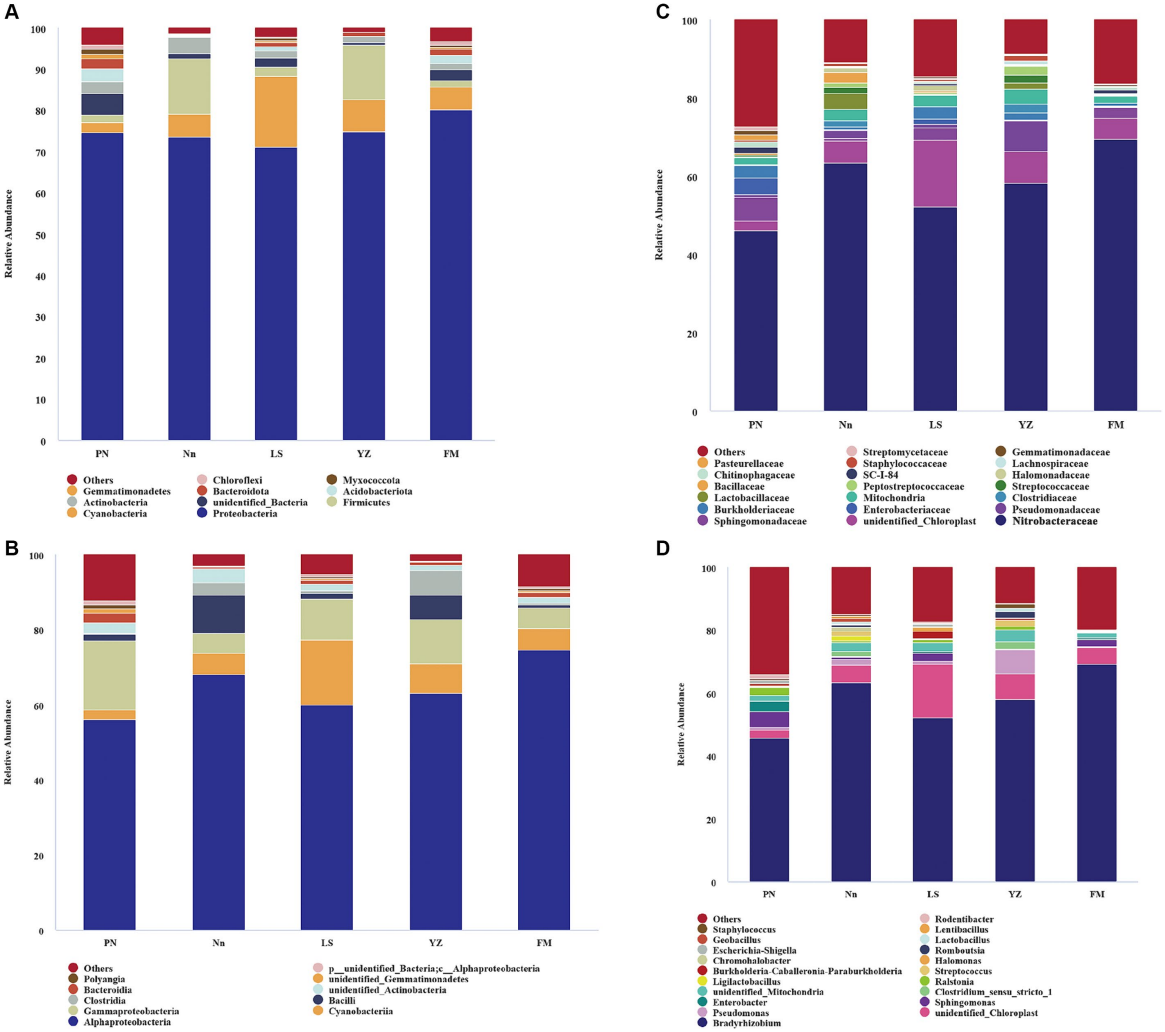
The relative abundance of bacteria at different levels. Bacterial groups at the phylum level **(A)**; bacterial groups at the class level **(B)**; bacterial groups at the family level **(C)**; bacterial groups at the genus level **(D)**. The analysis method is based on the relative abundance of species at the family and genus levels, using the ggplot2 package of R (v3.6.0) software to perform histogram analysis of species composition. Species with a relative abundance of less than 1% are represented by “others” in the legend.

**Figure 3:**
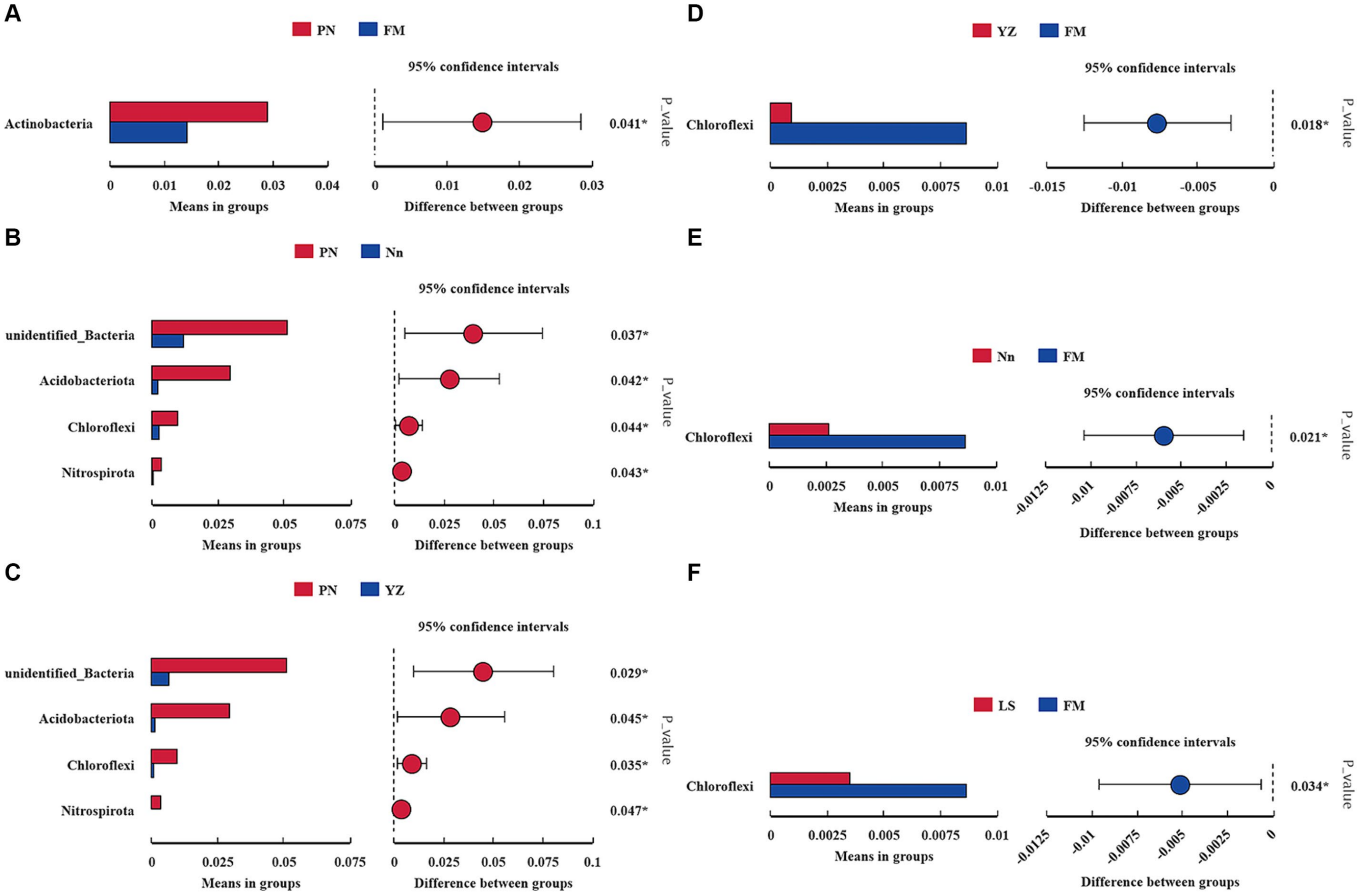
Comparison of the abundance of dominant bacterial genera in different samples. * indicates a significant difference at a *p* value < 0.05. The x-axis represents the mean proportion of the genus, and the y-axis shows the dominant bacterial genera. Comparison between PN and FM **(A)**. Comparison between PN and Nn **(B)**. Comparison between PN and YZ **(C)**. Comparison between YZ and FM **(D)**. Comparison between Nn and FM **(E)**. Comparison between LS and FM **(F)**.

The bacterial communities were highly diverse at the phylum level, with up to 47 bacterial phyla distributed among the five regions. The relative abundance of Proteobacteria was the highest in the five samples, accounting for 74.55, 73.49, 71.07, 74.72 and 80.24% in PN, Nn, LS, YZ and FM, respectively. The relative abundance of Cyanobacteria in PN (2.53%), Nn (5.65%), YZ (8.03) and FM (5.52%) was significantly lower than that in LS (17.19%). The relative abundance of Firmicutes in Nn (13.40%) and YZ (13.07%) was significantly higher than that in PN (1.92%), LS (2.22%) and FM (1.42%) (Figure 2A). Comparing the relative abundance of bacterial communities of all groups at the phylum level, some differences were observed (Figure 3). The relative frequencies of unidentified bacteria (*p* = 0.029), Actinobacteria (*p* = 0.045), Chloroflexi (*p* = 0.035) and Nitrospirae (*p* = 0.047) were higher in PN than in YZ. At the same time, the relative abundances of unidentified_Bacteria (*p* = 0.037), Actinobacteria (*p* = 0.042), Chloroflexi (*p* = 0.044) and Nitrospiota (*p* = 0.043) were significantly higher than those of Nn. The relative abundance of Actinobacteria (*p* = 0.041) was significantly higher in PN than in FM. In addition, no significant differences in PN were found for the other two groups at the phylum level. Chloroflexi had a significantly lower relative abundance in FM than in YZ (*p* = 0.018) and had a significantly lower relative abundance in FM than in Nn (*p* = 0.021) and LS (*p* = 0.034).

### 3.5 Microbial taxonomic analysis at the class and family levels

The bacterial communities were more diverse at the class level (Figure 2B). Moreover, the bacterial groups and the relative abundance of bacteria in root nodules from various sources were different. The bacterial community of PN mainly included 56.15% Alphaproteobacteria, 18.36% Gammaproteobacteria, 2.58% Bacteroidia, and 2.52% Cyanobacteria. The bacterial community of Nn was mainly composed of 68.07% Alphaproteobacteria, 10.22% Bacilli, 5.64% Cyanobacteria, and 5.43% Gammaproteobacteria. That of LS was mainly composed of 60.04% Alphaproteobacteria, 17.18% Cyanobacteria, and 11.03% Gammaproteobacteria. In YZ, 62.99% Alphaproteobacteria, 11.73% Gammaproteobacteria and 8.02% Cyanobacteria were observed.

There were differences in the composition of endophytic bacterial communities in root nodules from different sources at the family level, mainly in terms of relative abundance (Figure 2C). Specifically, the relative abundance of Xanthobacteraceae in PN (46.02%) was significantly lower than that in Nn (63.3%) and FM (69.37). The relative abundance of Sphingomonadaceae in PN, LS and FM was 5.98, 3.07 and 2.61%, respectively, and it was less than 1% in both Nn and YZ. The relative abundance of Enterobacteriaceae was 4.23% in PN and 1.28% in LS, while it was less than 1.00% in Nn, YZ and FM. Burkholderiaceae had a relative abundance of less than 1.00% in both Nn and FM.

### 3.6 Microbial taxonomic analysis at the genus level

The species evolutionary tree of the top 50 genera is shown in Supplementary Figure S2. These 50 bacterial genera belonged to eight phyla: Firmicutes (16 genera), Proteobacteria (14), Myxococcota (2), Verrucomicrobiota (1), Actinobacteria (11), Cyanobacteria (1), Bacteroidota (2) and Actinobacteria (3). Further analysis revealed that the endophytic bacterial communities showed high diversity at the genus level among root nodules from different sources (Figure 2D). The genus *Bradyrhizobium* was the dominant group in all samples, with a relative abundance of 45.79% in PN, 63.23% in Nn, 52.04% in LS, 58.14% in YZ and 69.17% in FM. The relative abundances of *Pseudomonas* in YZ and Nn were 7.76 and 1.99%, respectively, while they were less than 1.00% in Nn, PN and FM. The relative abundances of *Sphingomonas* and *Enterobacter* ranked second and third in PN, at 5.13 and 3.22%, respectively, well above those of the other groups. The relative abundance of *Ralstonia* in PN and YZ was 2.54 and 1.13%, respectively, but it was less than 1.00% in FM, Nn and LS.

These results show that the endophytic bacterial communities of root nodules from different origins had abundant diversity and that the dominant taxa were essentially the same. Root nodules in different regions tend to have rich endophytic bacterial communities.

### 3.7 Analysis of the influence of environmental factors on community structure and diversity

**Figure 4:**
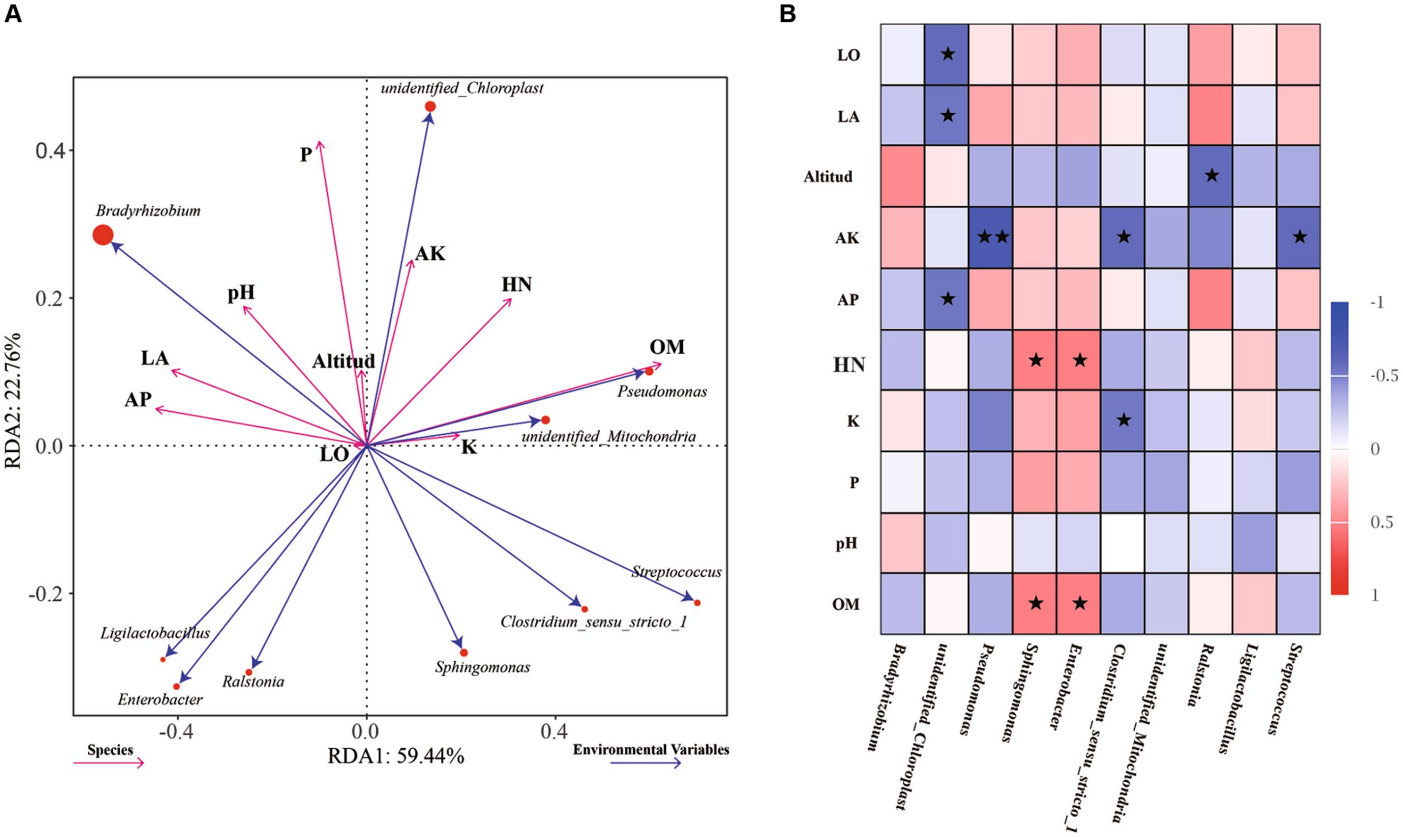
RDA of dominant endophytic bacteria and environmental factors of AC nodule samples: Environmental factors are indicated by arrows, with the lengths of the connection between the arrow and origin representing the degrees of correlation between environmental factors, community distribution and species distribution. The greater the length is, the stronger the correlation. The influence of environmental factors on bacteria is reflected by the cosine of the angle. The smaller the angle is, the stronger the correlation **(A)**. Correlations between the abundances of the top 10 genera and soil physicochemical and environmental factors were analyzed using Spearman’s correlation coefficient, * indicates significance at a *p* value <0.05, ^**^ indicates significance at a *p* value <0.01 **(B)**.

To further explore the influence of environmental factors on the endophytic bacterial community structure and diversity in the samples, the relationships between environmental factors and dominant bacteria were analyzed by RDA, with the soil physiochemical and environmental factors used as variables (Figure 4A). The effects of environmental factors on the endophytic bacteria in root nodules in the RDA diagram were characterized mainly by the length of the environmental factors, while the degree of influence on each strain was reflected by the cosine value of the angle. The content of organic matter (OM, *p* = 0.008) in soil was found to have significant effects on the endophytic bacteria in root nodules. The Spearman correlation coefficient was used to analyze the correlations of the top 10 genera with soil physiochemical and environmental factors (Figure 4B). The longitude and latitude of the environmental factors were negatively correlated with *Bradyrhizobium*, and the altitude was negatively correlated with *Ralstonia*. Available K (AK) and total K (K) were significantly negatively correlated with *Clostridium_sensu_stricto_1*, and AK was significantly negatively correlated with *Streptococcus* and very significantly negatively correlated with *Pseudomonas*. Available P (AP) was significantly negatively correlated with *unidentified_Chloroplast*. Hydrolysable N (HN) and organic matter (OM) were positively correlated with *Sphingomonas* and *Enterobacter*.

### 3.8 Rhizobial strain isolation and selection of representative strains

**Figure 5:**
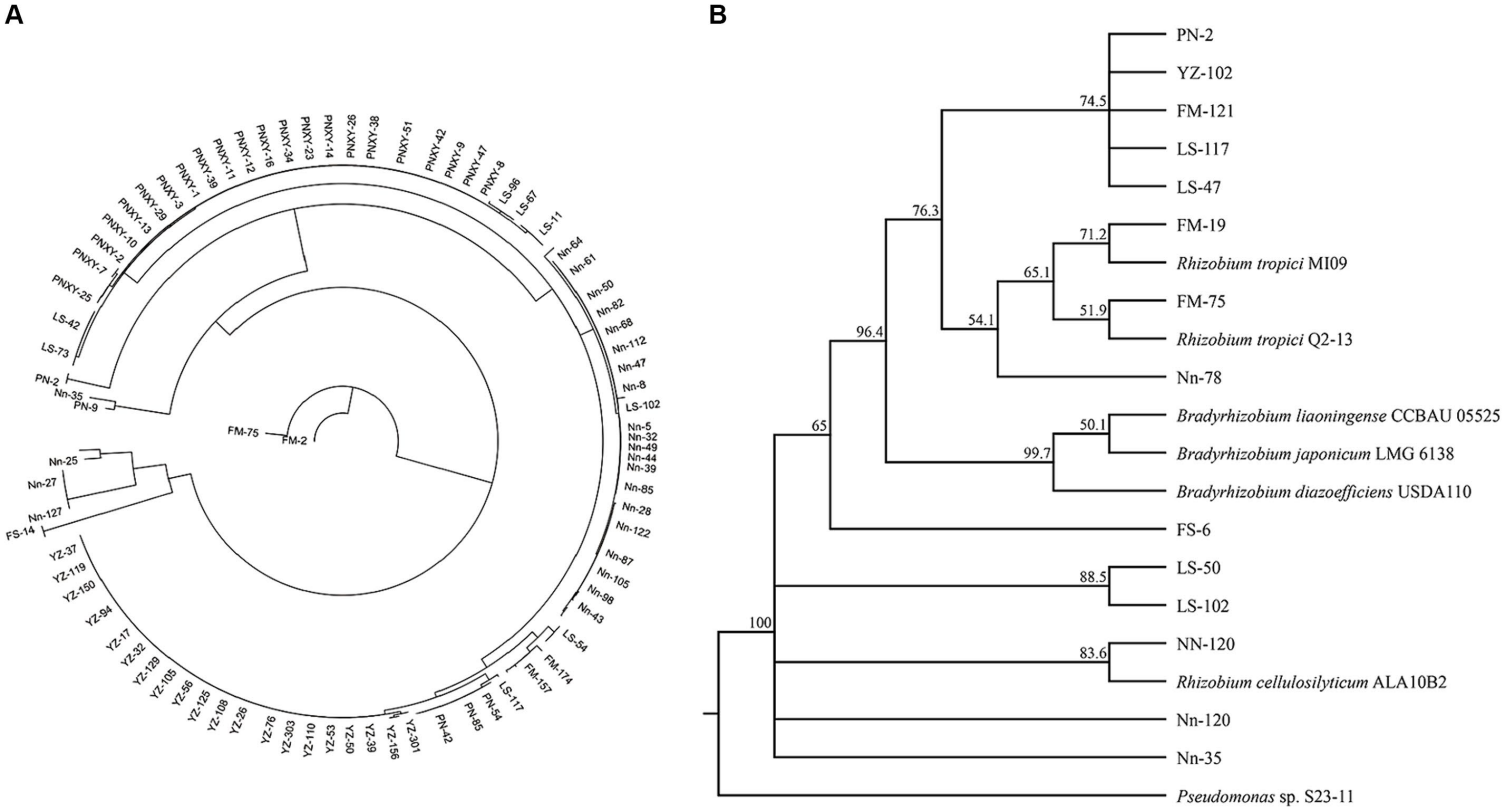
Neighbor-joining (NJ) phylogenetic tree based on *recA* gene sequences showing the clustering relationships of isolates **(A)**. Neighbor-joining (NJ) phylogenetic tree based on 16S rRNA gene sequences showing the relationships of isolated bacteria and reference strains (GenBank database). Bootstrap values (1,000 replicates) are indicated above the branches **(B)**.

A total of 179 (G^−^) purified strains were obtained from the five sampling sites after isolation and identification by Gram staining; the number of isolates obtained from the sites varied from 9 in FM to 58 in PN. Thirteen *rec* A haplotypes, among which one was found in only one strain and LS-47 was found in 61 strains, were classified according to *rec* A sequence analysis, and one randomly selected representative for each haplotype was used for the subsequent phylogenetic analyses (Figure 5A). The phylogenetic analyses of bacterial isolates were conducted by the alignment of 16S rRNA sequences using the BLAST search function in the GenBank database. The results in Figure 5B show that four rhizobial strains and nine rhizobial endophytic bacterial strains co-occurred in twelve haplotypes. The isolates FM-19 (PP325808) and FM-75 (PP325810) were grouped with *Rhizobium tropici*. The isolate Nn-120 was close to *Rhizobium cellulosilyticum* (PP325880).

### 3.9 Greenhouse experiment for the assessment of nodulation efficiency and plant growth

**Figure 6:**
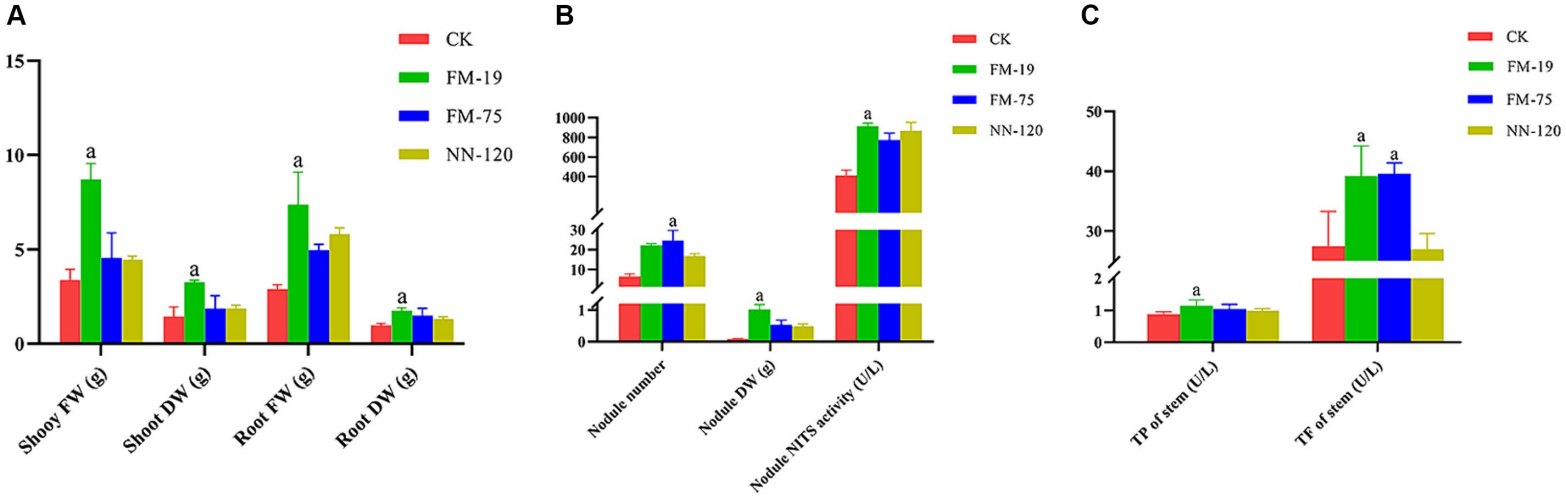
Plant parameters influenced by *Rhizobium* strain inoculation: shoot fresh weight (FW), shoot dry weight (DW), root fresh weight (FW) and root dry weight (DW) **(A)**; nodule number, nodule dry weight (DW) and nodule nitrogenase (NITS) activity **(B)**; total phenolics (TP) in stems and total flavonoids (TF) in stems **(C)**. The red column represents CK, green column represents FM-19 treatment, blue column represents FM-75 treatment and yellow-green column represents NN-120 treatment.

Rhizobial strain inoculation had significant positive effects on the growth and nodulation of AM plants compared to uninoculated controls 60 days after plant emergence. *Rhizobium tropici* FM-19 inoculation significantly enhanced the plant biomass, dry weight of nodules and nodule nitrogenase activity over those of the control. Interestingly, *Rhizobium tropici* FM-75 inoculation significantly enhanced the nodule number over that in the control, while nodules of plants subjected to FM-19 inoculation were fuller than those of FM 75-inoculated plants. Inoculation with FM-19 increased the shoot fresh weight (FW), shoot dry weight (DW), root FW and root DW of plants by 157.4, 126.5, 154.3 and 79.8%, respectively, compared with those of the uninoculated control (Figure 6A). In addition, the number of nodules, nodule DW and nodule nitrogenase activity increased by 98.0, 279.5 and 121.5%, respectively (Figure 6B). The total phenolic and total flavonoid contents of stems were increased by inoculation with *Rhizobium,* but this response was further enhanced with FM-19 application. The largest increase in total phenolics (30.9%) and total flavonoids (42.7%) was recorded for stems with FM-19 inoculation (Figure 6C).

## 4 Discussion

High-throughput sequencing provides a convenient and effective way to study the diversity of plant endophytes, which is conducive to the identification of low-abundance species and can reveal the structure of microbial communities in target environments in a comprehensive way and provide relevant biological information (Wang et al., 2021). However, traditional culture methods are still considered the main way to obtain target strains and show advantages in yielding endophytic bacterial resources with potential applications. For example, some endophytic bacteria with growth-promoting properties have been isolated by pure culture methods, and these endophytic bacteria can produce a variety of physiologically active metabolites, which have broad potential for development and application (Pavithra et al., 2022; Santos et al., 2022; Tori et al., 2023). Microbial endophytes play an important role in promoting the ecological functions, health, and growth of plants, especially *Rhizobium*, which can facilitate symbiotic nitrogen fixation. However, there are limited reports on the endophytic microbial populations present in AM nodules. Therefore, in this study, Illumina MiSeq was used to sequence the V3-V4 variable region of the bacterial 16S rRNA gene to study the diversity of endophytic bacteria in AM nodules. A total of 179 rhizobial isolates were isolated from root nodules of AM grown at the 5 sampling sites. Liu et al. (2021) found that *recA* could be widely used to define rhizobial haplotypes. We defined *recA* gene sequences with 100% sequence similarity as the same genotype and categorized the isolated bacteria into 13 groups. The 16S-derived data reflect a predominance of *Bradyrhizobium* with more than 50% in almost all samples. However, *Bradyrhizobium* was not isolated in our results. During the incubation, by the third day of incubation, colonies had grown all over the plate with the minimum concentration of suspension. So we had to culture and preserve these single colonies in pure form in advance, which may have contributed to the fact that we did not obtain *Bradyrhizobium*. As stated by the experts, they need at least 3–4 days to form colonies during sub-culturing, and even longer when isolated from the wild. Because they need at least 3–4 days to form colonies during successional cultures, and longer if isolated from the field.

The results showed that the symbiotic nitrogen-fixing rhizobia present within the nodules mainly belonged to *Rhizobium*, *Ensifer*, *Bradyrhizobium*, *Mesorhizobium*, *Azorhizobium* and *Methylobacterium* in Alphaproteobacteria and to *Burkholderia* and *Cupriavidus* in Betaproteobacteria. The nonnitrogen fixing bacteria were mainly from *Bacillus*, *Pseudomonas*, *Enterobacter*, *Chryseobacterium* and *Sphingobacterium* (Mouradi et al., 2016). Some of the endophytic bacteria detected in the AM nodules in this study were the same as those found in the other plant endophytes mentioned above, especially at the phylum level, and were similar to those found in soybean nodules (Akifumi et al., 2017), which mainly contain the phyla Proteobacteria, Firmicutes, Actinobacteria, and Bacteroidetes, suggesting that the endophytic bacteria present in AM nodules were somewhat similar to those present in the nodules of other plants in terms of their community composition. Many *Bradyrhizobium* have been isolated from other legume hosts and subsequently named after the host plant. For example, Ramírez-Bahena et al. (2009) isolated *Bra. pachyrhizi* from nodules of yams; Chahboune et al. (2011) isolated *Bra. cytisi* from *Cytisus villosus*; and Wang et al. (2013) isolated a new species, *Bra. daqingense*, from soybean. In this study, the 16S-derived data reflect a predominance of *Bradyrhizobium* with more than 50% in almost all samples. The dominant phylum in the AM nodules collected from five different locations was Proteobacteria because Proteobacteria are able to utilize a wider variety of substrates than other bacteria (Chao et al., 2015). However, in terms of environmental factors, we found opposite trends between Proteobacteria and Firmicutes, suggesting that Proteobacteria and Firmicutes have opposite environmental preferences, which is consistent with the findings of Zhang et al. (2018). These nonrhizobial endophytes are considered opportunists since they can infect nodules when rhizobia induce nodule formation (Leite et al., 2017). In addition, the predominance of nonrhizobial isolates was attributed to the genus *Pseudomonas*. This result is consistent with the findings of Cardoso et al. (2018), who showed that the genus *Pseudomonas* is one of the most dominant genera in several legume nodules. *Pseudomonas* is commonly classified as a plant bacterial endophyte and can reduce Fusarium root rot and promote soybean growth (Dengqin et al., 2023).

The root system is the main organ in plants for absorbing water and nutrients, and inoculation with *Rhizobium* promotes root system growth and the absorption and utilization of nutrients and water by the root system. Rasheeda et al. (2023) showed that inoculation with rhizobial strains significantly increased the root length and root volume of *Vigna radiata* compared to a control, and similar findings were obtained in the present study. Plant inoculation with rhizobial strains increases photosynthetic capacity and improves water and nutrient absorption, which ultimately results in an increase in plant biomass. The symbiotic interaction between AM and *Rhizobium* is the result of mutual selection over a long period of evolution and adaptation to the environment. In this study, we showed that AM inoculation with three different strains of rhizobia under the same conditions did not have the same growth effect, although all of the rhizobia could symbiotically colonize AM and perform nitrogen fixation. This result suggests differences in symbiotic matching between rhizobia and host plants (Igolkina et al., 2019). The full capacity for symbiotic nitrogen fixation and the promotion of plant growth and active ingredients can be achieved only by the selection of suitable strains. The rhizobia isolated in this study were from different regions, each with its own habitat. Although AM was the host for all these rhizobia, there were genotypic differences in the AM grown in different regions, and thus, inoculation under the same conditions produced inconsistent results. With *Astragalus sinicus,* the inoculum match and nitrogen fixation capacity of different rhizobia also varied considerably (Burghardt et al., 2017). Nine strains of rhizobia belonging to three species were isolated and obtained in this study and were identified as *Rhizobium tropici* and *Rhizobium cellulosilyticum*. In greenhouse experiments, *Rhizobium tropici* exhibited strong growth-promoting properties and positive effects on total flavonoid and total phenol accumulation in AM. Banuelos et al. (2023) found that *Rhizobacteria tropici* could secrete riboflavin, which affects AMF symbiosis and promotes the growth of *Phaseolus vulgaris* L. Similarly, Diez-Mendez et al. (2015) found that *Rhizobium cellulosilyticum* as a coinoculant enhances *Phaseolus vulgaris* grain yield under greenhouse conditions. Notably, both *Rhizobacteria tropici* and *Rhizobium cellulosilyticum* were first identified in AM and show strong growth promotion in AM, which has a large impact on the crop management of AM. However, multisite field trials are required to demonstrate the success of this technology under natural field conditions.

## 5 Conclusion

In this study, the bacterial diversity and composition of AM nodules were elucidated for the first time using high-throughput sequencing methods. The content of OM in soil was found to have significant effects on endophytic bacteria in AM nodules. *Bradyrhizobium* was the dominant genus in all samples, and among the environmental factors, longitude and latitude were negatively correlated with *Bradyrhizobium*. In addition, the results of a greenhouse experiment demonstrated that the *Rhizobium tropici* FM-19 isolated in this study had a highly significant growth-promoting effect on AM and could significantly increase the accumulation of total phenols and total flavonoids in AM plants.

## Data availability statement

The data presented in the study are deposited in the NCBI Short Read Archive (SRA) database repository, accession number PRJNA1091308.

## Author contributions

KC: Data curation, Writing – original draft. JC: Methodology, Writing – review & editing. QL: Investigation, Writing – review & editing. PG: Visualization, Writing – review & editing. LL: Conceptualization, Funding acquisition, Writing – review & editing. RH: Conceptualization, Funding acquisition, Writing – review & editing.
