# Supplementary material for: Bacteria from nodules of Abrus mollis Hance: genetic diversity and screening of highly efficient growth-promoting strains
Source: Front Microbiol. 2024 Apr 12;15:1345000. doi: 10.3389/fmicb.2024.1345000 (PMC11045970; doi:10.3389/fmicb.2024.1345000)

Supplementary Figure S1: Venn diagram of the OTU distribution of the endophytic bacterial community detected in root nodules taken from five different locations. Notes: Different colors represent different samples. The overlapping areas of circles of different colors represent common OTUs, and unique OTUs are present in the nonoverlapping parts.

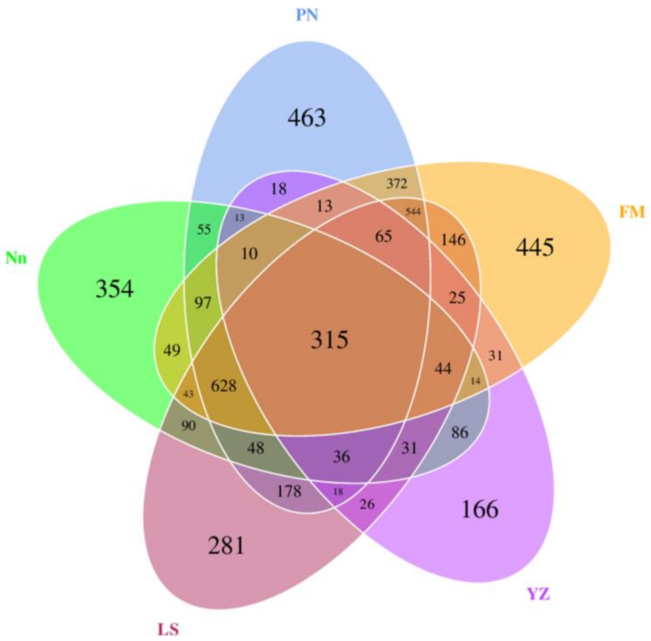

Supplementary Figure S2: Phylogenetic tree at the genus level constructed via multiple sequence alignment. The middle of the phylogenetic tree shows the evolutionary classification of the species, the colors of the branches indicate corresponding phyla, each color represents one phylum, and the bars in the outer circle represent samples with the highest proportion of the genus and their abundance.

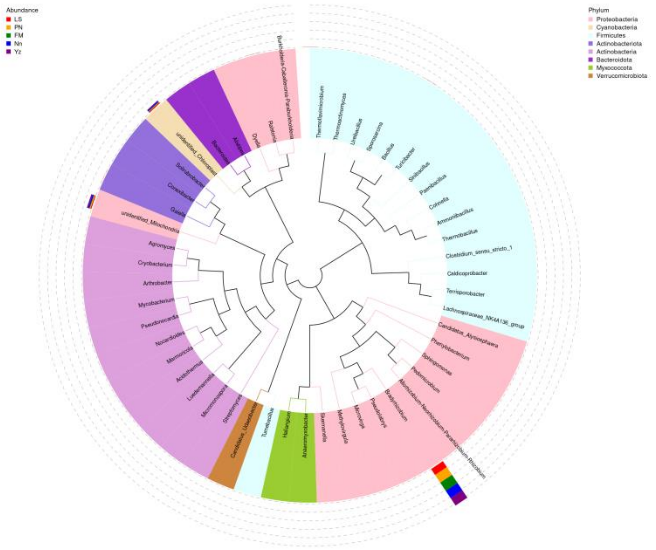

Supplement: Supplementary file 1 [file Image_1.pdf]
